# A Vectorial Current Density Imaging Method Based on Magnetic Gradient Tensor

**DOI:** 10.3390/s23135859

**Published:** 2023-06-24

**Authors:** Yangjing Wu, Mingji Zhang, Chengyuan Peng, Zehuang Zhang, Yichen He, Wenwei Zhang, Liang Chang

**Affiliations:** 1 Sino-German College of Intelligent Manufacturing, Shenzhen Technology University, Shenzhen 518118, China; yangjing.wu@foxmail.com (Y.W.); 2070412005@stumail.sztu.edu.cn (C.P.); zehuang.zhang@foxmail.com (Z.Z.); yichen_he@foxmail.com (Y.H.); zhangwenwei@sztu.edu.cn (W.Z.); 2 State Grid Liaoning Electric Power Co., Ltd., Huludao 125000, China; ln_hld_sgcc@126.com

**Keywords:** magnetic current imaging, magnetic gradient tensor, current density inversion, closed-form inversion, nondestructive testing

## Abstract

Magnetic current imaging is deemed an emerging powerful technique for visualizing electrical currents in electronic devices. However, the existing magnetic-field-based Fourier Transform back-evolution method is limited by its mono-function of imaging the magnitude of current density in devices under test, and subject to background noise distortion. Here, we developed a novel vectorial current density imaging method based on the detection of the magnetic field gradient generated by current carrying conductors. A closed form solution of current density inversion was analytically derived and numerically verified. Experiments were conducted by scanning tri-axial fluxgate sensor over different shapes of electrical wires. The results show that a current density resolution of 24.15 mA/mm^2^, probe-to-sample separation of 2 mm, and spatial resolution of 0.69 mm were achieved over a maximum scanning area of 300 mm × 300 mm. Such a method is verified to be capable of simultaneously imaging both magnitude and directions of current density, which is a promising technique for in situ noninvasive inspection for the power electronic and semiconductor industry.

## 1. Introduction

The current flows in power electronic devices not only reflect the electromagnetic compatibility, joule heating sources, and impedance performances of devices under test (DUT), but also determine the fault hazard, reliability, as well as stability of the electronic system.

State-of-the-art electronic device detection relies on lumped parameter measurements of the current–voltage (I–V) curve, impedance spectrum, contact resistance and *S* parameter, etc., by connecting the test probe to the circuit net or node. These lumped parameters are believed to be useful in identifying the running status of DUT, but are uncapable of indicating the abnormal reason and fault position.

**Figure 1:**
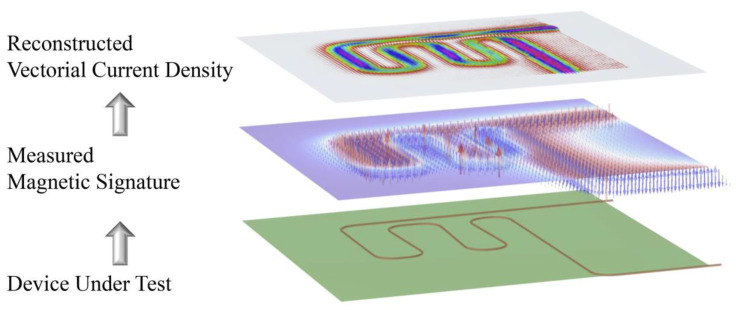
Schematic diagram of vectorial current density imaging based on magnetic signature.

Magnetic current imaging (MCI) is a powerful measurement tool to visualize the current flows in DUT by non-invasively sensing the magnetic signature [1]. This novel technique provides a spatial distribution of the current density (CD) in an image manner to quantitively and straightly localize abnormal positions, as illustrated in Figure 1.

Magnetic current imaging was firstly proposed for bio-magnetic detection, and applied in an integrated circuit test by Bradley J. Roth and E. F. Fleet, respectively [2,3]. Existing MCIs primarily use Fourier transform back-evolution for the vertical component of the magnetic field above the DUT to reconstruct its two-dimensional CD magnitude distribution [4,5]. The superiorities of in situ, visibility, and non-invasive conditions promoted MCI applications in the fields of integrated circuit fault diagnosis, superconductor magnet analysis, and lithium-ion battery health prognosis [1,6,7,8]. 

Benaiah D. Schrag et al. used magnetic tunnel junction sensors to image the CD magnitude in a self-made application-specific integrated circuit. Current and spatial resolutions of 1 mA and 1 mm, respectively, were achieved over probe-to-sample separation of a 4 mm scanning area of 2 × 2 mm^2^. This work discussed the imaging quality dependence on measurement noise and separation distance, however, lacked a technical solution [9]. J. Gaudestad et al. implemented a *μ*m-level resolution of CD and successfully localized the die level short-circuit used in giant magneto resistance sensors [6]. Pauli Kehayias et al. imaged the surface current density magnitude in 555 timer IC by measuring magnetic fields using a quantum diamond microscope, achieving the highest micron-scale spatial resolution and few-micro-Tesla magnetic sensitivity in a 1 × 1 μm^2^ pixel at a probe-to-sample separation of 26 μm [10]. M. Sumi and N. Satoh obtained the CD contribution on a Cockcroft–Walton circuit, a Schottky rectifier and a multi-layered ceramic capacitor. An electric short circuit spot was successfully observed by magneto-impedance sensor within an operation area of 50 × 50 mm^2^ [11]. Mark G. Bason et al. achieved internal CD magnitude imaging in lithium-ion batteries using fluxgate sensor arrays within a scanning area of 150 cm^2^ to give a spatial resolution of 5 mm [7]. Felix Brauchle et al. demonstrated the discovery of cuts and erroneous weld spots inside lithium-ion cells using anisotropic magnetic resistance sensors with a current resolution of 0.15 mA, with a probe-to-sample separation of 7.8 mm. On the other hand, the accuracy of CD in this work is 227 mA/cm^2^ at a local resolution of 4 mm^2^ [12]. Their CD imaging results intuitively distinguished CD pattern differences between a normal and faulty status, however, were unable to offer CD directions [13]. E. Marchiori et al. showed the CD in the superconducting qubit circuit using a superconducting quantum interference device (SQUID) to give a resolution of 3 mA and a probe-to-sample separation of 600 nm [8]. 

The emergence and development of MCI enable a new insight to reveal the essential electrical property of the DUT. However, the latest MCI technique is limited in providing CD magnitude pattern, and uncapable of offering vectorial current density (VCD) information, which is of great significance in deriving divergence and rotation signatures to identify current leakage and an equivalent magnetic induction. Moreover, the magnetic field is the fundamental information for a CD reconstruction algorithm; therefore, the magnetic noise will finally couple to the inversed CD results and determine the accuracy and quality of current density images. Filtering, signal windowing, and magnetic shielding were adopted to suppress the equivalent magnetic noise [14,15,16,17], however, were inapplicable in reducing the common mode noises arising from background electrical equipment and earth magnetic fluctuation [18,19].

In this paper, a VCD imaging method is proposed to simultaneously image the VCD distribution based on tensorial magnetic signature, and reduce the common mode background noise using a second-order gradient technique [20,21]. A closed-form vectorial current density inversion method is established by combining the difference approximation of differentials and the second-order gradient method. Square, triangular, circle, and snake shapes of current-carrying wires are experimentally adopted as typical DUTs and were vectorially current-imaged using a tri-axial fluxgate sensor. FEA models of the above DUTs are correspondently developed to give coherent VCD images between numerical results and experimental results, with a relative error less than 10%. Although this work is implemented in 16AWG electrical wires, the VCD reconstruction method is fully applicable to other types of current carriers such as superconductor wires, silicon-based chips, and power batteries, etc. It is expected to be migrated to areas of semiconductor, electronic, and electrical energy industries as novel tools for design, characterization and health monitoring. VCD’s promising ability to identify current leakage will greatly facilitate the online inspection of electronic products. 

## 2. The Closed-Form Vectorial Current Density Inversion Method

The magnetic field, H, generated by an electrical current in a conductor is governed by the well-known Ampere Law, of which the differential form can be written as:

(1) ∇×H=J

where ∇× is the curl operation, and J the current density vector determined by the cross-section and total current flow in the conductor. 

By applying the linear constitution relationship of *B–H* in air: $B=\mu_{0}H$, the current density can then be reformulated as:

(2) $$J=\frac{1}{\mu_{0}}\left(\nabla \times B\right)=\frac{1}{\mu_{0}}\left|\begin{array}{ccc} e_{x} & e_{y} & e_{z}\\ \frac{\partial }{\partial x} & \frac{\partial }{\partial y} & \frac{\partial }{\partial z}\\ B_{x} & B_{y} & B_{z} \end{array}\right|=\frac{1}{\mu_{0}}\left[\begin{array}{c} \left(\frac{\partial B_{z}}{\partial y}-\frac{\partial B_{y}}{\partial z}\right)e_{x}\\ \left(\frac{\partial B_{x}}{\partial z}-\frac{\partial B_{z}}{\partial x}\right)e_{y}\\ \left(\frac{\partial B_{y}}{\partial x}-\frac{\partial B_{x}}{\partial y}\right)e_{z} \end{array}\right]$$

where B is the magnetic flux density, $\mu_{0}$ the vacuum permeability, and $e_{x}$, $e_{y}$, and $e_{z}$ the unit vector for a Cartesian coordinate system, respectively. Equation (2) indicates that the vectorial current density, J, is measurable as long as the spatial variation of the magnetic flux density, B, can be obtained. Applying the physical definition of the magnetic field gradient, $G_{ij}=\partial B_{i}/\partial j$, to Equation (2), the vectorial current density, J, can be linearly expressed in a closed form as a function of the magnetic field gradient tensor elements, described as:

(3) $$J=\left[\begin{array}{c} J_{x}e_{x}\\ J_{y}e_{y}\\ J_{z}e_{z} \end{array}\right]=\frac{1}{\mu_{0}}\left[\begin{array}{c} \left(G_{zy}-G_{yz}\right)e_{x}\\ \left(G_{xz}-G_{zx}\right)e_{y}\\ \left(G_{yx}-G_{xy}\right)e_{z} \end{array}\right]$$

### 2.1. Measurement of the Magnetic Field Gradient 

**Figure 2:**
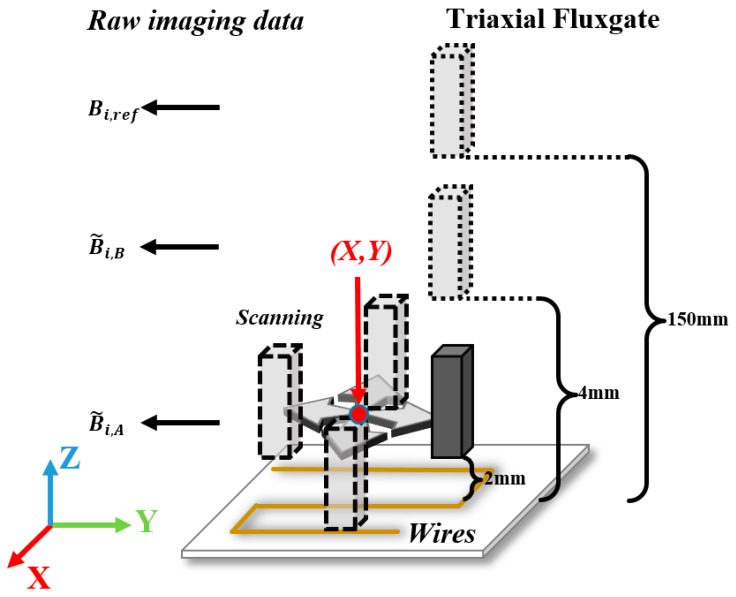
Schematic of the second-order magnetic field gradient measurement system.

As shown in Figure 2, the theoretically defined magnetic field gradient in Equation (3), mathematically also the spatial differential of magnetic flux density **B**, can be experimentally obtained by spatial differencing $B_{i,A}$ and $B_{i,B}$ over the baseline ($L_{j}$), given by:

(4) $$G_{ij}=\frac{B_{i,A}-B_{i,B}}{L_{j}},\;\left(i,j=x,y,z\right)$$

where $B_{i,A}$ and $B_{i,B}$ are common-mode noise-rejected magnetic flux density components at each sampling position obtained by point-by-point background noise ($B_{i,\mathrm{ref}}$) subtraction, described as:

(5) $$B_{i,A}={\tilde{B}}_{i,A}-B_{i,\mathrm{ref}}\;\mathrm{and}\;B_{i,B}={\tilde{B}}_{i,B}-B_{i,\mathrm{ref}}$$

### 2.2. Raw Imaging Data Formatting

**Figure 3:**
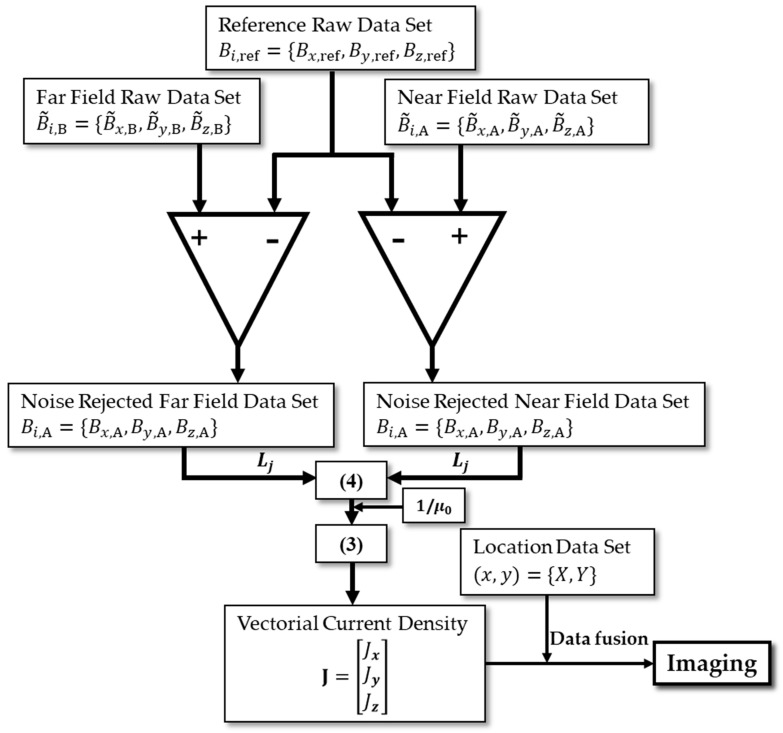
Schematic of the raw imaging dataset processing diagram.

Through the data measuring process shown in Figure 2, we can get a near-field raw dataset, far-field raw dataset, and reference dataset. Figure 3 illustrates the data flow and processing diagram for a vectorial current density imaging method based on the magnetic gradient tensor. First, a struct buffer is applied to store background noise as a reference raw dataset. Second, far-field and near-field tri-axial magnetic flux density datasets are differenced with respect to the reference raw dataset, respectively, to give a noise-rejected far-field and near-field dataset. Third, the second-order differencing is conducted with respect to baselines ($L_{j}$) to offer a magnetic field gradient dataset. Then, the VCD dataset can be attained based on Equation (3). Finally, by spatially fusing the VCD dataset and location dataset point by point, the vectorial image data of current density are precepted and recorded in an RGB format, where R, G, and B quantitively represent the *x*, *y*, and *z* components of CD. It is practically necessary to mention that each component, as well as the vector strength, can be visualized through a pseudo-color image (e.g., rainbow or traffic cloud picture) with a combination of arrows.

## 3. Experimental Setup

**Figure 4:**
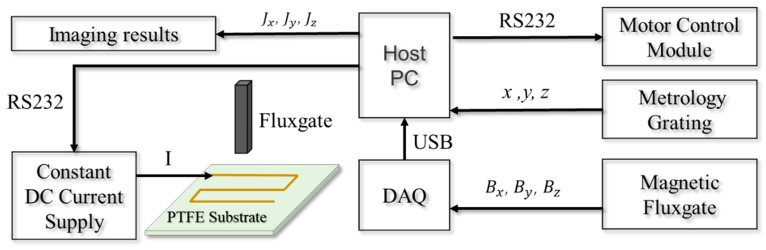
Schematics diagram of the experiment setup for VCD imaging.

**Figure 5:**
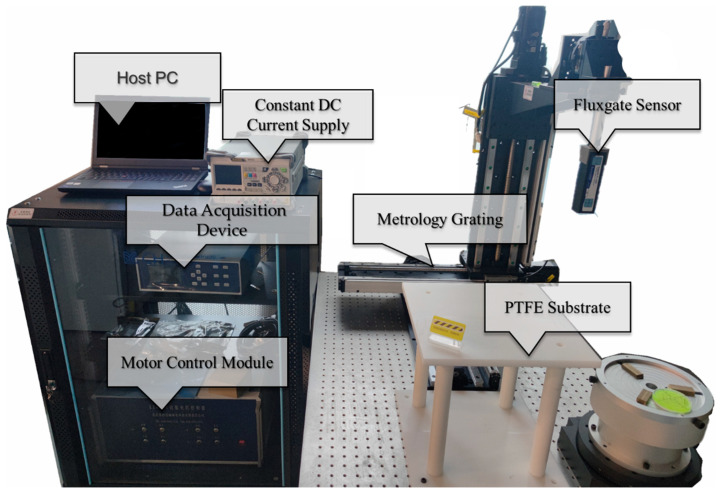
Vectorial current density imaging system based on magnetic gradient tensor measurement.

**Figure 6:**
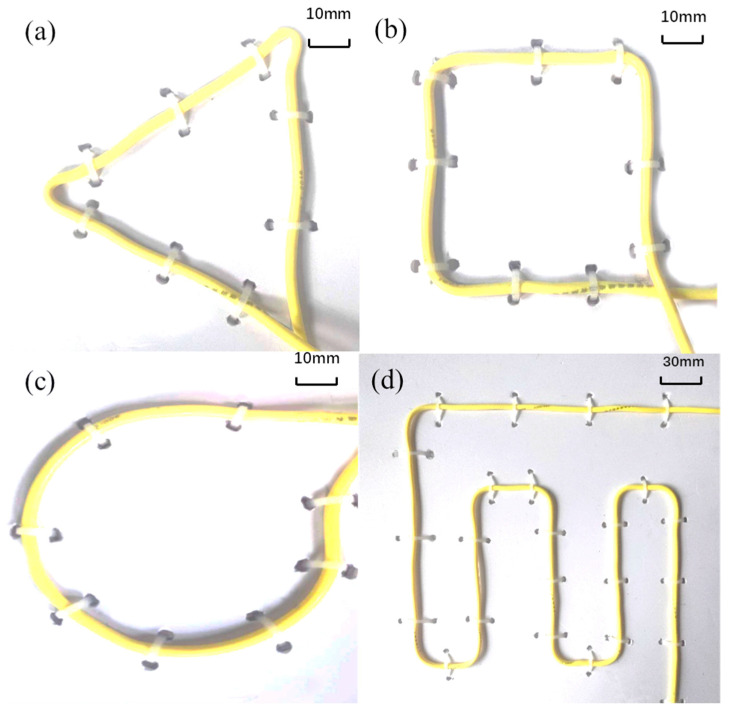
Various shapes of DUTs. (**a**) Triangle; (**b**) square; (**c**) circle; (**d**) snake shape.

Figure 4 shows the schematic diagram of the experiment setup for the closed-form vectorial current density inversion method. The experiment system (Figure 5) consists of seven parts: the non-magnetic DUT substrate, metrology grating module, step-motor control module, host PC, DC current supply, data acquisition device, and a tri-axial fluxgate sensor. Four typical DUTs, namely square, triangular, circle, and snake shapes, made of 16AWG electrical wires (Figure 6) are excited by the DC constant current of 5A fed by a power supply (RIGOL^®^ DP831A, Beijing, China). The DUTs are horizontally fixed on a PTFE substrate with a size of 300 mm × 300 mm. The motor control module (CH-Magnetoelectricity^®^ Technology KZ400C, Beijing, China) communicates with the host PC through an RS-232 protocol with synchronous location data measured via metrology grating. Triaxial magnetic field data at the reference, near, and far positions are precisely measured using a tri-axial fluxgate sensor (Bartington^®^ Mag-690, Oxon, UK), and recorded by a data acquisition device (CH-Magnetoelectricity Technology^®^ CH370, China). The imaging data processing program illustrated in Figure 3 is embedded in a host PC to display the VCD picture. A spatial resolution of 0.01 mm within range of 400 mm, and a magnetic field resolution of 0.1 nT within a range of ±100 *μ*T were declared on the manufacture datasheet. 

## 4. FEA Modeling

A series of FEA models are developed strictly following the dimensions and shapes of each DUT, as shown in Figure 6, to calculate theoretical magnetic flux densities around DUTs. The FEA models are established on the COMSOL Multiphysics platform (COMSOL^®^ Co., Ltd., V5.5, Gothenburg, Sweden), using an AC/DC module by coupling an electric current solution into an external current source, as in Ampere’s Law governing equation. Constant current terminal and ground boundary conditions are applied on the feeding and end boundaries of DUTs, respectively. No flux boundary condition is applied on the ball-shape air domain. Cubic discretization is selected to guarantee the accuracy of second-order magnetic gradient results. Magnetic flux densities from FEA solutions are numerically extracted from two 2 mm separated parallel planes to form theorical results of far-field and near-field dataset. The simulation results of the reconstructed VCD image can be eventually obtained following the same process as shown in Figure 3. Such an FEA model has three functions of providing ‘exact’ CD results to check experimental–theoretical errors, obtaining ‘exact’ magnetic fields and its gradient patterns to investigate error coupling effects during the reconstruction algorithm, and analyzing the differences between numerical and experimental VCD images. 

## 5. Results and Discussion

**Figure 7:**
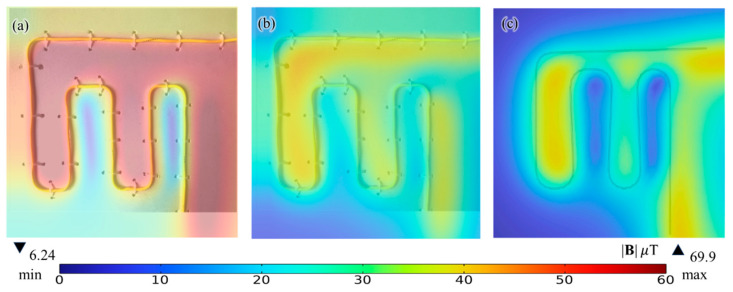
Magnetic flux density strength |**B**| @ 2 mm above the DC-5A excited snake shape conductor of (**a**) near-field raw data; (**b**) noise-rejected near-field data; (**c**) FEA simulation data.

Figure 7 shows the raw near-field, noise-rejected near-field, and numerical magnetic flux density |**B**| images @ z = 2 mm. The magnetic fields are generated by a DC-5A excited snake shape conductor to represent most DUTs without losing its generosity. We see from Figure 7a that the vector superposition of magnetic fields from DUTs and background magnetic fields are measured to give a relatively high magnetic flux density |**B**| up to 69.9 *μ*T. Figure 7b,c shows that the maximum magnetic flux density |**B**| @ z = 2 mm is evaluated to be 41.7 *μ*T, 41.7 *μ*T, and 42.1 *μ*T from Biot–Savart’s solution, numerical simulation and experimentally noise-rejected data, respectively. The results indicate that a highly coherent magnetic field results with a relative error less than 1% can be obtained using a differencing technique with respect to the reference raw dataset based on Equation (5). 

**Figure 8:**
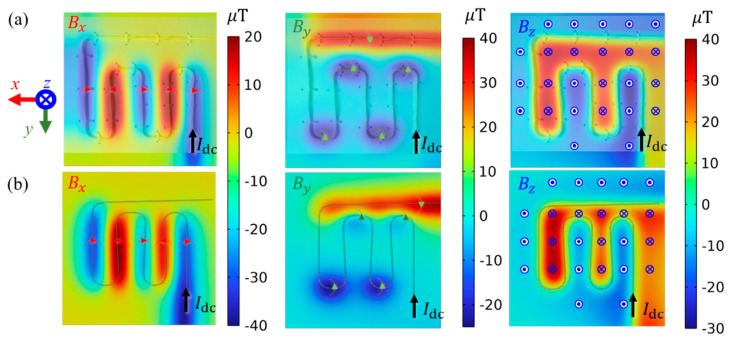
Vectorial magnetic flux density **B** @ 2 mm above the DC-5A excited snake shape conductor of (**a**) noise-rejected near-field experiment results; (**b**) FEA simulation results.

Figure 8a,b shows the *x*, *y*, and *z* components of experimentally noise-rejected near-field data and simulated near-field data in Figure 7b,c correspondingly. Following the positive directions of the imbedded Cartesian coordinate, the feeding DC current from the bottom end of DUT results in obvious $B_{x}$ above *y*-guided wire, $B_{y}$ above *x*-guided wire, and $B_{z}$ for all wires are governed by famous ‘right-hand rule’. In comparison with magnetic flux density |**B**| in Figure 7, the components of **B** intuitively reflect the directions of CD, however, cannot directly offer current flow directions, especially when a non-orthogonal condition occurs between wire directions and **B** directions. Benefiting from excellent noise rejection performance endowed using a differencing technique, highly coherent results are obtained between experiment and simulation results, as shown in Figure 8a,b, respectively. It can be also found that the $B_{z}$ image can roughly indicate the wire contours through its cross-zero isogram. This may be the fundamental principle of traditional Fourier Transform back-evolution method for MCI [2]; however, it is limited in providing the magnitude of CD only. 

**Figure 9:**
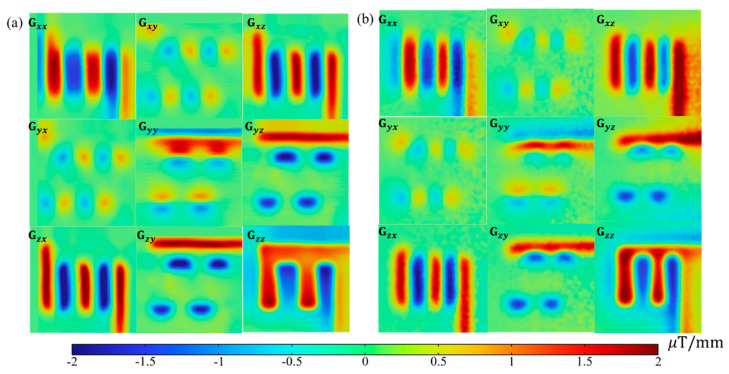
Snake-shape DUT induced magnetic field tensor elements of (**a**) experiment results; and (**b**) simulation results.

Figure 9a,b shows the full tensor of magnetic field gradient images of experiments and simulation results, respectively. As the most critical intermediate variable, the full magnetic tensor images are derived from measurement results in Figure 8 according to Equation (4). An intuitive impression of large zero areas (colored green in the rainbow bar) leaps to the eyes from the tensor images. This can be attributed to the effects of second-order operations, which has exempted magnetic features induced by non-local CD. Moreover, both experiment and simulation results exhibited a high coherence and strictly symmetric pattern ($G_{ij}=G_{ji,\;}\;i\neq j,\;i,j=x,y,z$), which are intrinsic properties of the magnetic tensor. Such property can be practically used as data check criteria. It is also interesting to find that the non-zero value of $G_{xy}$ or $G_{yx}$ appears on the corner. Further examination indicates the right-hand rotation CD results in positive $G_{xy}$, and vice versa. Such a discovery is not applicable to $G_{xz}$ and $G_{yz}$ because the scanning is conducted parallel to the *x–y* plane. 

**Figure 10:**
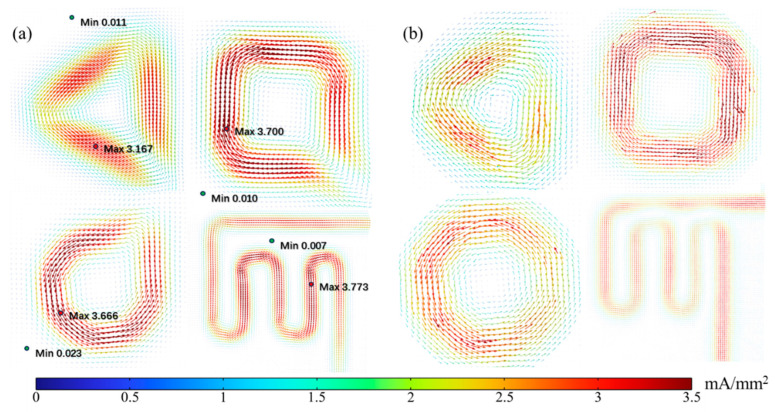
Reconstructed VCD images of DC-5A excited DUTs. (**a**) Experiment results; (**b**) simulation results.

Figure 10a,b shows the reconstructed vectorial current density imaging results based on the measured and simulated magnetic gradient tensor, respectively, based on the closed form inversion formula Equation (3). A relative error of less than 3% is achieved between experiment results and simulation results for all shapes of DUTs. The CD magnitude and its directions are successfully imaged with obvious evidence to distinguish different shapes of DUTs. Through the closer examination of the experimental VCD image, the ingoing and outgoing imprints can be recognized as shown in Figure 10a. The difference between the calculated CD under operating condition result (|**J**| = 5 A/1.5 mm^2^ ≈ 3.33 A/mm^2^) and reconstructed result |**J**| = 3.5 mA/mm^2^ can be explained through a probe-to-sample distance induced lift effect. That is, magnetic field attenuated third order inversely with respect to sensing distance, resulting in a smaller CD magnitude and diffusive CD images. The CD magnitude attenuation can be corrected by multiplying a fixing factor (*k* = 1050 in this case); however, the diffusion effect can only be alleviated by reducing the probe-to-sample distance. The overall CD resolution is estimated to be 24.15 mA/mm^2^ by selecting the maximum of the best resolutions among the four DUT CD experimental images, and by multiplying a fixing factor of 1050. It is worthy to mention that the CD resolution can be improved by simply reducing the probe to sample distance. The spatial resolution is estimated to be 0.69 mm with respect to the wire radius. It is expected to improve the spatial resolution by reducing the size of the sensor to image the CD in a printed circuit board or even a chip. 

## 6. Conclusions

We have analytically proposed, numerically realized, and experimentally verified a novel current density imaging method with the simultaneous advantages of providing current flow directions and suppressing common-mode noise. A spatial resolution of 0.69 mm and current density resolution of 24.15 mA/mm^2^ were achieved, with a probe-to-sample separation of 2 mm over a maximum scanning area of 300 mm × 300 mm. The intermediate variables of magnetic flux density **B**, components, and tensor are incidentally imaged and interpreted to reveal the magnetic signatures around current carriers. We also point out that reducing the size and improving sensitivity are the most efficient technical routes to promote both the spatial resolution and current resolution of magnetic vectorial current density imaging. It is expected that the reconstructed vectorial current density will provide new electrical information which can be applied in areas of PCB, IC, and superconductor inspection. 

## Data Availability

The data presented in this study are available on request from the corresponding author. The data are not publicly available due to some funding policies.
